# Primary ciliary dyskinesia and psychological well-being in adolescence

**DOI:** 10.1371/journal.pone.0227888

**Published:** 2020-01-23

**Authors:** Selene Valero-Moreno, Silvia Castillo-Corullón, Inmaculada Montoya-Castilla, Marián Pérez-Marín

**Affiliations:** 1 Department of Personality, Assessment and Psychological Treatments, Faculty of Psychology, University of Valencia, Valencia, Spain; 2 Pediatric Pneumology Unit, Hospital Clinico Universitario de Valencia, Valencia, Spain; Hong Kong Polytechnic University, HONG KONG

## Abstract

Primary ciliary dyskinesia (PCD) is a rare autosomal recessive disease with low prevalence in pediatrics. Health studies have not sufficiently analyzed the role of psychological variables in rare diseases such as PCD. This paper studies the psychological characteristics of a group of pediatric patients diagnosed with PCD compared to their healthy peers. The sample consisted of 48 preadolescents-adolescents, aged 9–18 years (M = 12.96; SD = 2.71), with similar distribution by sex, and 25% of the patients having dyskinesia. Clinical anxiety-depression, self-esteem and psychological well-being were evaluated using questionnaires: the Adolescent Psychological Well-being Scale (BIEPS-J), the Hospital Anxiety and Depression Scale (HADS) and the Rosenberg Self-Esteem Scale (RSE). Data were analysed using descriptive, mean comparison (t-test) and diffuse comparative qualitative analysis (QCA). The results show no differences were found between healthy and PCD patients in the variables analyzed, except for social ties showing the latter greater well-being in this aspect. In QCA models, the variables that best explained the high or low levels of well-being were depression and self-esteem, and primary ciliary dyskinesia was not a necessary condition for presenting low levels of well-being. In conclusion, our results highlight the need to explore psychological aspects in pediatric patients with rare diseases.

## Introduction

Primary ciliary dyskinesia syndrome (PCD) is an inherited, autosomal recessive disease, considered rare due to its low prevalence[1–3]. It is characterized by chronic upper and lower respiratory tract infections, including the middle ear, from birth[4,5]. Bronchiectasis is present in more than 80% of patients[6], leading to recurrent and chronic lung damage. The involvement of scourges of both male and female reproductive cells is what predisposes patients to infertility, a characteristic feature of this pathology[6]. Studies indicate that 1/15,000–20,000 live births[7] will present the disease, with a similar distribution among boys and girls[2,8]. The average age at diagnosis of PCD is approximately 4 years[1].

Despite medical advances, there is currently no treatment to correct ciliary dysfunction in PCD. Most treatments applied to patients with PCD are similar to those used with other similar diseases, such as cystic fibrosis[1,6,9].

In paediatrics, research on the psychological aspects (well-being, internalizing- externalizing problems, self-esteem) of chronic respiratory diseases is scarce, especially as related to rare diseases such as PCD. Uncertainty about the prognosis and evolution of the disease, ongoing medical care, as well as a lack of symptom control, often negatively impact these patients’ emotional well-being[10–12]. Infertility is also an important characteristic associated with PCD, which can lead to frustration or negative emotions in them and their families due to its possible consequences for the patient's future plans [13].

Research on chronic pediatric disease indicates a greater presence of clinical anxiety-depression in these patients compared to their healthy peers[12–15] more behavioural problems[15–17] and a worse perceived psychological well-being[15,18]. In addition, these patients appear to report lower self-esteem[19–21]. This aspect is particularly important in adolescence, when self-esteem seems to be an important protector associated with psychological well-being[21], facilitating adaptation to this stage of life and to possible present illnesses, reducing emotional suffering and the appearance of complications[21].

Due to the paucity of research specifically examining these aspects in primary ciliary dyskinesia, this paper studies the psychological characteristics of a group of pediatric patients diagnosed with PCD in comparison with their healthy peers.

## Method

This research involving human participants has been approved by the Ethics Committee from the University of Valencia Review Board (IRB) (References: (H1435211034634), and conducted according to the principles expressed in the Declaration of Helsinki. Written Informed consents have been obtained from the legal guardians of the participants.

### Participants

The study sample consisted of 48 pre-adolescents (9–12 years old) and adolescents (13–18 years old). They were aged between 9–18 years, with a mean age of 12.96 years *(SD = 2*.71). This total sample is divided into two subsamples: 1) pre-adolescents and adolescents with PCD, 2) pre- adolescents and healthy adolescents.

The subsample of healthy individuals (with no chronic physical health problem) was 36 subjects (19 men and 17 women; age = 12.78±2.50) from several education centres of Valencia. 50% were preadolescents (n = 18) and the remaining 50% were adolescents (n = 18).

The PCD subsample consisted of 12 patients (7 men and 5 women; age = 13.50 years ±3.34) diagnosed at least 6 months previously, and receiving regular out-patient healthcare at the Pneumology Service of the Hospital Clínico Universitario de Valencia (a leading Unit in PCD in the Valencian Community). 41.7% were preadolescents (n = 5) and the rest 58.3% were adolescents (n = 7).

### Instruments

#### (a) Sociodemographic and medical variables

Ad hoc patient records were developed, containing the following information: age, sex, months since diagnosis and in treatment, type of daily treatment, other secondary diagnoses, number of hospitalizations during the lifetime, and spirometry values.

#### (b) Psychological variables

The *Psychological Well-Being Scale for Adolescents* (BIEPS-J)[22] based on Ryff's Multidimensional Psychological Well-Being Model. This measures psychological well-being on four subscales: situation control, psychosocial bonds, self-acceptance and projects. It consists of 13 items, with 3 answer options: "agree", "neither agree nor disagree" and "disagree". It has an overall emotional well-being score, which is the total of all the scores. In the original study, the internal consistency of the scale obtained an alpha score of .74 for the overall questionnaire[22]. In addition, suitable psychometric properties were found with adolescents[23]. The following averages are found in the Spanish sample: control 10.61, psychosocial bonds 8.50, projects 7.47, self- acceptance 7.44 and total psychological well-being 34. Based on these, the equal or higher (high well-being) and lower (low well-being)[24] cut-offs were established.

The *Rosenberg Self-Esteem Scale* (RSE)[25] version adapted to the Spanish context by Atienza, Balaguer and Moreno[26]. It consists of 10 items (a Likert format, ranging from 1 -Strongly disagree, to 4—Strongly agree), focused on feelings of respect for and acceptance of oneself. The original study[25] obtained an internal consistency of .92. The alpha scores in Spanish studies of adolescents have a reliability of .86[27]. The total score ranges from 10 to 40 points, distinguishing between low (scores less than or equal to 29) and high (equal to or greater than 30) self-esteem.

The *Hospital Anxiety and Depression Scale* (HADS)[28] developed as a screening instrument for identifying non-psychiatric patients with affective disorders attending hospitals[29]. The items evaluate cognitive clinical anxiety and depression, as opposed to the somatic clinical profile[29], a relevant issue in studies with subjects with a significant medical/somatic diagnosis. It is divided into two dimensions: the anxiety subscale (HADS-A) and the depression subscale (HADS-D). Adding the scales of anxiety and depression provides an overall score for emotional distress. The questionnaire has been used in adolescents and young people[30] (from 10 to 23 years old), and validated for pediatric samples[31], obtaining adequate psychometric properties[31,32]. Scores between 0–6 represent no anxiety, 7–9 anxiety possible, over 10 anxiety probable. In depression, 0–5.4 represent no depression, 5.5–7.5 depression possible, over 7.5 depression probable and for emotional distress, below 15.5 no emotional distress, and over 15.5 emotional distress probable[33].

### Procedure

The data was collected at the Hospital Clínico Universitario de Valencia. First, patients with PCD were identified (inclusion criteria: more than 6 months since diagnosis, aged between 9–18 years, regularly attending the pediatric pneumology clinic in the hospital. Exclusion criteria: a) infantile cerebral palsy (ICP), b) Brain tumours, c) Attention deficit or hyperactivity disorder (ADHD), d) psychological diagnosis prior to the onset of respiratory diagnosis).

The data was collected by members of the research team, both at the hospital and at schools in the Valencian Community, in a single pass and at a single point in time. Informed consent was requested from the legal guardians of the participants, who were informed of the characteristics of the study, assured of the commitment to confidentiality, and informed about the recording of personal data. We were endorsed by the ethical committee of the University of Valencia and the INCLIVA Foundation (H1435211034634), and had obtained the relevant permits from the Spanish Ministry of Education.

### Data analysis

Mean and standard deviations were calculated for the variables analysed and recoding according to cut-off points, and t-tests were performed to analyse mean differences between groups using the SPSS statistical programme. The raw data from the participants' responses had to be transformed for QCA. First, as suggested in the literature[34], all the missing data were eliminated and all conditions (variables) were calculated by multiplying the scores of their items. Before performing the analysis, the values had to be recalibrated between 0 and 1. When we consider only two values, we use 0 (does not have the characteristic) and 1 (has the characteristic). With continuous variables or with factors from a survey, it was necessary to consider the following three thresholds: 10% (low-level or totally outside the set), 50% (intermediate level, neither inside nor outside the set), and 90% (high level or totally inside the set). These three values had to be introduced in order to perform an automatic re-calibration of values between 0 and 1. In this case, the fsQCA 3 software[34] re-calibrated the values for psychological variables considering the three thresholds[35]. QCA models are based on a Boolean or intersection logic, and assume the influence of a particular attribute or attributes on a specific outcome(equifinality).

## Results

### Medical variables

The months since diagnosis of patients with PCD amounted to around 5 years (M = 55.45 months; SD = 35.15 months). The months in medical treatment were similar (M = 61.82 months; SD = 41.51 months). The number of hospitalizations during the lifetime was 3.90 (SD = 4.36), and 75% of patients were followed up approximately every 3 or 4 months. The daily dose (deemed to be the total number of tablets, inhalations or nebulizations per day) was 2.75 (SD = 1.42). 83.3% therefore received inhaled therapy (aerosols), 50% nebulized therapy and 58.3% were taking pills (antibiotics, oral corticosteroids, etc.). 91.7% of the cases of PCD were other adjacent respiratory pathologies (bronquiectasis, inhalant allergies, asthma, malaceous) with the most predominant being bronchiectasis (75%). Of the 8 patients with bronchiectasis, 4 were cylindrical, 3 varicose and 1 both, and a single patient also presented an additional non-respiratory diagnosis such as celiac disease. No patient had pseudomonal infection. and only one had situs inversus.

Meanwhile, the spirometry values recorded during the evaluation indicated that 25% of patients showed a mild obstructive pattern. The data collected were as follows: Forced Vital Capacity (FVC) averaged 106.25%, with values ranging from 95.8–131.1%. For forced expiratory volume in the first second (FEV1), an average of 99.21% (82.1–123.4%) and the mean ratio between FEV1/FVC was 81.89% (65.49–92.96).

### Psychological variables

#### Psychological well-being

In the subsample of healthy adolescents (subsample HA), 41.7% had poor interpersonal ties, 33.3% had a lack of vital projects and 36.1% had low acceptance of themselves. Interestingly, 41.7% presented low overall psychological well-being. Finally, as shown in Table 1, in pediatric patients with PCD, 50% presented problems of situation control, 58.3% a lack of vital projects, 25% a low acceptance of themselves, and 8.3% had poor interpersonal ties. Finally, 41.7% presented low overall psychological well-being. As Table 2 shows, the only variable in which differences between the two samples were observed was interpersonal ties (t = -2.18; *p* = .04). Patients with PCD showed better links than their healthy peers.

**Table 1:** Total recoded well-being scores for subsamples (HA and PCD).

BIEPS-J variables	Healthy susbsample (n = 36)		PCD subsample (n = 12)	
	Low well-being	High well-being	Low well-being	High well-being
**Control**	52.8	47.2	50	50
**Interpersonal Ties**	41.7	58.3	8.3	91.7
**Projects**	33.3	66.7	58.3	41.7
**Acceptance**	36.1	63.9	25	75
**Total Well-being**	41.7	58.3	41.7	58.3

**Table 2:** Results of means comparisons of psychological variables in PCD patients and healthy.

Questionnaire	Variables	Healthy (n = 36) *M*^a^ (*SD*^b^)	PCD^c^ (n = 12) *M* (*SD*)	t value	*Df*^d^	*p*^e^	*d*^f^ *[95% interval confidence)*	*F*^g^ ratio	*p variances*
**BIEPS-J**^h^	Control	10.19(1.51)	10.67(1.56)	-0.94	46	.36	0.32 [0.10–0.74]	0.08	.78
	Interpersonal ties	8.28(1.16)	8.83(0.58)	-2.18	38.62	.04	0.54 [0.24–0.83]	4.93	.03
	Projects	7.83(1.30)	6.83(1.99)	1.63	14.25	.13	0.68 [0.21–1.10]	5.52	.02
	Acceptance	7.61(0.15)	8.25(1.06)	-1.40	46	.17	1.22 [1.08–1.38]	1.19	.28
	Total well-being	33.92(3.74)	34.58 (3.90)	-0.53	46	.60	0.18 [-0.87–1.23]	0.09	.76
**HADS**^i^	Anxiety	5.61(4.43)	6.42 (4.93)	0.42	13.84	.68	0.18 [-1.08–1.44]	4.14	.05
	Depression	3(2.38)	2.50 (3.89)	-0.53	14.71	.61	0.19 [-0.60–0.96]	4.26	.05
	Emotional distress	8.61(4.76)	8.92(7.99)	-0.13	13.69	.90	0.06 [-1.52–1.63]	9.86	.001
**RSE**^j^	Self-esteem	31.31(5.72)	31.9(4.54)	-0.35	46	.74	0.11 [-1.40–1.62]	0.52	.47

^a^ mean

^b^ standard deviation

^c^ Primary ciliary dyskinesia

^d^ degrees of freedom

^e^ level of significance

^f^ d de Cohen effect size ES small ≈ 0.20; ES moderate ≈ 0.50; ES large ≈ 0.80

^g^ F-ratio test was applied for differences of variance between groups calculation

^h^ Psychological Well-Being Scale for Adolescents

^i^ Hospital Anxiety and Depression Scale

^j^ Rosenberg Self-Esteem Scale

#### Self-esteem

**Fig 1:**
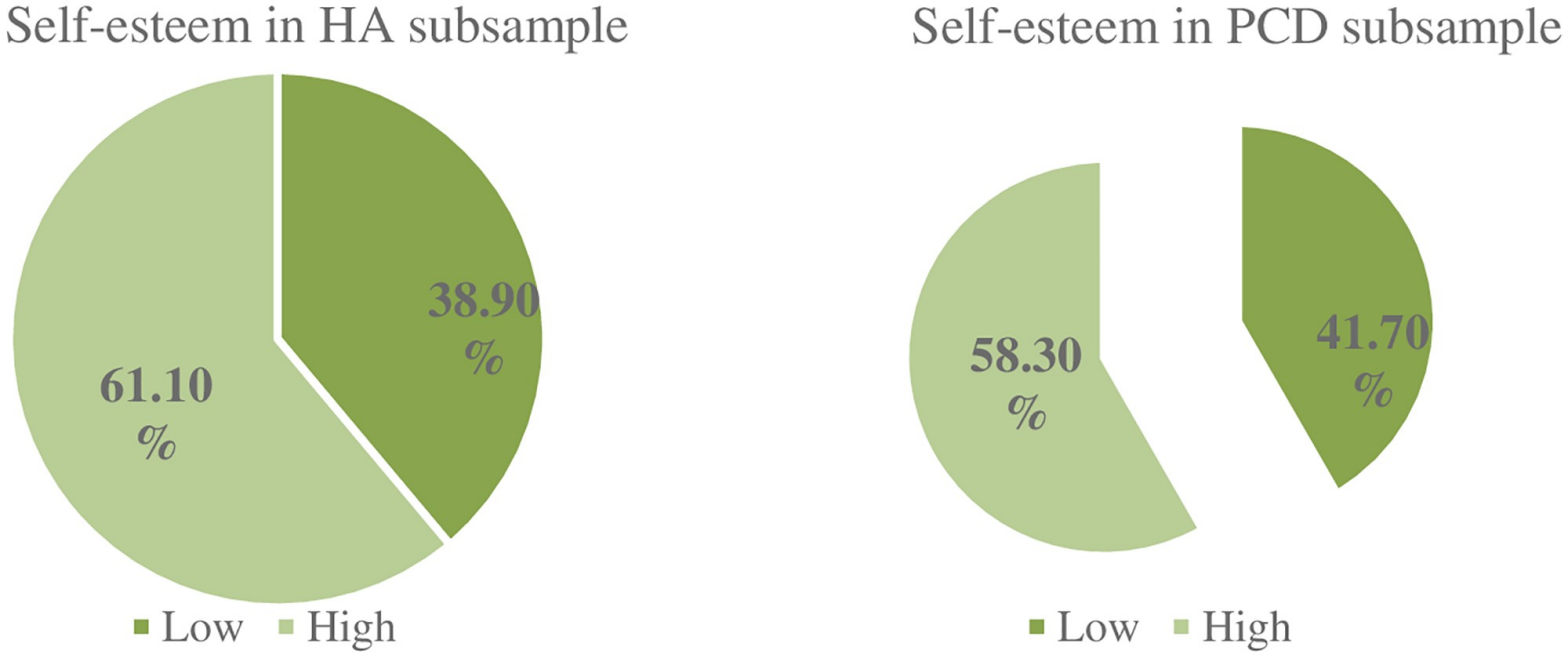
Recoded total self-esteem scores for each of the subsamples.

The scores obtained for self-esteem are shown in Fig 1. In the HA sub-sample, 38.9% had low self-esteem. and in the PCD sub-sample, the figure rose to 41.7%. No differences in self-esteem were found between the two groups, as shown in Table 2.

#### Emotional distress

In the HA sub-sample, 33.3% presented symptoms of anxiety, with 13.9% having a problem of clinical anxiety. In depression, 5.6% presented a clinical problem of depression. Finally, 11.1% suffered from a clinically significant problem of emotional distress.

In the PCD sub-sample, 41.7% presented symptoms of anxiety, which was especially important, as 25% presented a clinical problem of anxiety. In depression, the mean was lower but clinically significant, as 16.7% had a clinical problem of depression. 33.3% suffered from a clinically significant problem of overall emotional distress (Table 3). Although no significant differences for any of the variables were observed between the groups (Table 2), patients with PCD descriptively presented higher scores for anxious symptomatology, while healthy participants did so for depressive symptomatology.

**Table 3:** Total scores of anxiety, depression and emotional distress recoded for each sample.

Variables HADS^a^	Healthy susbsample (n = 36)			PCD^b^ subsample (n = 12)		
	Normal-absence	Probable case	Clinical problem	Normal-absence	Probable case	Clinical problem
Anxiety	66.70	19.40	13.90	58.30	16.70	25
Depression	86.10	8.30	5.60	83.30	-	16.70
Emotional distress	88.90	-	11.90	66.70	-	33.30

^a^ Hospital Anxiety and Depression Scale

^b^ Primary ciliary dyskinesia

#### Qualitative comparative analysis of diffuse sets (QCA)

First, as the literature suggests, need analyses were performed (see Table 4), followed by sufficiency analyses (see Table 5). “Psychological well-being" was established as the criterion variable (outcome condition), and sex, age, presence of PCD, self-esteem, anxious and depressive symptomatology as predictor variables (causal conditions).

**Table 4:** Main descriptions and calibration values.

	Anxiety	Depression	Self-esteem	Psychological well-being
*M*^a^	141.77	15.48	193031.37	443398.50
*SD*^b^	282.88	33.82	269349.55	486098.17
Min^c^	1	1	32	972
Max^d^	1536	216	1048576	1594323
P10^e^	2	1	1101.60	6609.60
P50^f^	28	4	62208	314928
P90^g^	576	33.6	786432	1594323

^a^mean

^b^standard deviation

^c^ mínimum

^d^maximum

^e^10th percentile;

^f^50th percentile

^g^90th percentile.

**Table 5:** Necessary analysis for emotional well-being.

	High levels of well-being		Low levels of well-being	
	Cons.^a^	Cov.^b^	Cons.	Cov.
Girl	.44	.40	.47	.60
Boy	.56	.43	.53	.57
PCD^c^	.27	.45	.24	.55
Without PCD	.73	.41	.76	.59
Teen	.55	.45	.50	.55
Pre-teen	.45	.39	.50	.61
High self-esteem	.35	.76	.21	.64
Low self-esteem	.84	.44	.92	.66
High anxiety	.55	.56	.54	.76
Low anxiety	.76	.54	.69	.68
High depression	.50	.46	.63	.80
Low depression	.79	.61	.58	.62

^a^ Consistency: Necessary condition: consistency ≥.90

^b^ Coverage

^c^ Primary ciliary dyskinesia

### Necessary analysis

Low self-esteem is the only necessary condition for the low levels of emotional well-being of pre-adolescents and adolescents (Table 5), because its consistency was greater than 90.[36]

### Sufficiency analysis

The resulting models for each dimension (based on the assumption that in QCA a model is informative when the consistency is around or above .74[37] provided the following results: in the prediction of high levels of psychological well-being, five pathways were observed that explained 59% of the cases with high levels (total consistency = .82; total coverage = .59). The most relevant pathways for predicting high levels of well-being were the result of the interaction of low levels of depressive and anxious symptomatology, high self-esteem and the absence of PCD (raw coverage = .26, consistency = .90). The second path was the interaction of low levels of depressive and anxious symptomatology, being an adolescent, having no illness and being a girl (raw coverage = .16, consistency = .83); and finally the combination of high anxious symptomatology, being an adolescent, not having PCD and being a boy (raw coverage = .12, consistency = .79) (Table 6).

**Table 6:** Summary of the three main conditions sufficient for the intermediate solution of psychological well-being.

Frequency cut-off 1	High well-being Consistency cut-off .79			Low well-being Consistency cut-off .80		
	1	2	3	1	2	3
Girl		^a^ ● ^a^	**◯** ^b^	●		**◯**
PCD^c^	**◯**	**◯**	**◯**		**◯**	
Teen		●	●		**◯**,	**◯**
High self-esteem	●			**◯**	**◯**	**◯**
High anxiety	**◯**	**◯**	●			**◯**
High depression	**◯**	**◯**		●	●	
Raw coverage	.26	.16	.12	.30	.27	.16
Unique coverage	.15	.08	.10	.13	.04	.05
Consistency	.90	.84	.79	.93	.88	.79
**Overall solution consistency**			.**82**			.**85**
**Overall solution coverage**			.**59**			.**65**

^a^presence of condition

^b^ absence of condition.

^c^ Primary ciliary dyskinesia

Expected vector for high levels of well-being: 0,0,0,1,0,0,0 (according to Fiss nomenclature, [38]. Expected vector for low levels of well-being: 1,0,1,0,1,1.

Meanwhile, for the prediction of low levels of psychological well-being, six pathways were observed that explained 65% of the cases with low levels (total consistency = .85; total coverage = .65). The most relevant combinations were the result of the interaction of high depressive symptomatology, low self-esteem and being a girl (raw coverage = .30, consistency = .93), which explained 30% of the cases with low levels. The second combination was the interaction between high depressive symptomatology, low self-esteem, being pre-adolescent and not having PCD (raw coverage = .28, consistency = .89), which explained 28% of the cases, and finally the combination of low anxious symptomatology, low self-esteem, being pre-adolescent and being boy (raw coverage = .16, consistency = .79), which explained 16% of the cases.

## Discussion

The diagnosis of a rare disease in a child has a major impact on the child and his or her parents. In relation to PCD, uncertainty about the prognosis[8], possible future medical complications[1,9] and associated fertility problems, especially in boys[2,6], lead to an increase in worries and a loss of the sensation of control in adolescent patients, reducing their emotional well-being.

With this objective in mind, we examined the role that psychological factors can play in pre-adolescence and adolescence. At the same time, we have assessed the possible weight that a chronic medical pathology such as PCD can have on these individuals, comparing PCD patients with their healthy peers as in previous studies[12,15,18].

The results obtained for well-being indicate that the sample of patients with PCD shows worse scores in projects compared to their healthy peers (having to follow daily treatment regimes and the sensation of uncertainty about the future can lead them to be included in fewer future plans). However, patients with PCD do not present worse rates of psychological well-being than their healthy peers, as indicated by previous studies [15,18]. One interesting variable is that patients with PCD have more social bonds. Most patients with PCD report good interpersonal relationships. This is particularly important, given that adolescence is a period of searching for interpersonal growth and independence. Having the support of the reference group such as the peer group can act as an important protective factor in the process of adaptation to the disease[12], this requires further study in future research. Overall, we observed that a percentage of pre-adolescents and adolescents show low levels of well-being, which is an aspect requiring attention in possible work with them.

Although the literature suggests that at this stage disease can reduce levels of self-esteem[19], with self-esteem being a positive variable in the patient's adjustment, no differences were found between healthy and PCD. Nevertheless, this vital period of changes can have a negative impact on their image of themselves, since approximately 40% of the whole sample showed low levels of self-esteem. Second, with regard to emotional symptomatology, the predominant psychopathological clinical feature is anxiety in PCD, which is most characteristic in respiratory diseases[10,16]. Uncertainty about the future, fears (of deterioration, respiratory exacerbations, fertility problems in adulthood, etc.) can increase levels of anxiety in this type of patients. In addition, the frustration of normal expectations[13] and having to adapt their daily routines to time-consuming treatments (between 2 or 3 hours a day) can also have a negative impact on emotional well-being, leading to the appearance of a depressive clinical profile, as shown in our results and those of previous studies[10]. We found no differences between patients with PCD and their healthy peers, as compared to other studies[12,13,15], and observed that depressive symptomatology is more present in healthy people and anxious symptomatology in PCD. In this case, psychological support is necessary to improve emotional well-being.

Previous studies suggest that a chronic condition may be a risk factor in adolescents’ adjustment and psychological well-being. The results found using QCA models show that PCD does not appear to be a variable that influences emotional well-being[15]. One possible explanation is that patients with PCD are stable, i.e. they maintain a good lung capacity, which means that they are not limited in their activities of daily living, such as physical activities, leading to a better well-being.

Other variables such as self-esteem and anxiety-depressive symptomatology therefore appear to be associated with levels of well-being. High self-esteem with low symptomatology or no presence of anxiety and depression would this explain the high levels of well-being. Depression and self-esteem are therefore the main variables that would account for levels of well-being. The role of the gender and age variables (pre-adolescent-adolescent) is not fully clarified, and further studies are necessary.

An analysis of the role of psychosocial variables in rare pediatric diseases (such as PCD) is important due to the paucity of studies on this subject. This study explores a field in which the number of investigations is currently meagre[15,18]. It is not without some limitations—the number of patients with PCD is limited, although all these patients present a fully confirmed diagnosis and are not only diagnostic suspicions. All patients who met the inclusion criteria were recruited. The limited number of patients and the single-center nature of this study may have altered the results and a research with a wider, nationwide population can end up with different conclusions, although in future research it would be appropriate to continue to expand the sample. Meanwhile, the use of self-reports such as HADS can often lead to social desirability bias due to underestimating responses. In future research, it would therefore be appropriate to compare these results with other informants such as parents, or with objective data such as spirometry values, but it was statistically difficult in this study due to the number of patients. Further research based on the results we have obtained may help to increase social awareness of the importance of psychological interventions in pediatric patients’ process of adjustment to rare diseases.

## Conclusions

Our results highlight the need to explore psychological aspects in pediatric patients with rare diseases. It is essential for healthcare professionals specializing in the care of these patients to have in-depth knowledge of the psychological characteristics associated with primary ciliary dyskinesia. Knowing which variables are associated with the psychological health of patients can help reduce the negative impact on their emotional well-being and improve their medical care.

## Supporting information

S1 Data set Healthy_PCD. (DAT)
